# Real-world Rollout of Injectable Antiretrovirals for HIV Prevention and Treatment: Correlates of Early Adoption

**DOI:** 10.1093/ofid/ofaf029

**Published:** 2025-01-20

**Authors:** Liza Koshy, Erika Payne, Lydia Barakat, Ritche Hao, Soundhari Sureshanand, Andrea Cedillo Ornelas, Andrew Dewan, Jaimie P Meyer

**Affiliations:** Chronic Disease Epidemiology, Yale School of Public Health, New Haven, Connecticut, USA; Yale New Haven Health, New Haven, Connecticut, USA; Infectious Diseases, Yale School of Medicine, New Haven, Connecticut, USA; Infectious Diseases, Yale School of Medicine, New Haven, Connecticut, USA; Yale Center for Clinical Investigation, Joint Data Analytics Team, New Haven, Connecticut, USA; Yale School of Medicine, New Haven, Connecticut, USA; Chronic Disease Epidemiology, Yale School of Public Health, New Haven, Connecticut, USA; Chronic Disease Epidemiology, Yale School of Public Health, New Haven, Connecticut, USA; Infectious Diseases, Yale School of Medicine, New Haven, Connecticut, USA

**Keywords:** antiretroviral therapy, HIV, implementation, long-acting injectable, PrEP

## Abstract

**Background:**

Data are limited on implementation of long-acting injectable (LAI) HIV treatment (ART) and preexposure prophylaxis (PrEP). We characterized “early adopters” of LAI ART and PrEP in terms of social determinants of health using a health equity lens.

**Methods:**

Our retrospective cohort included patients prescribed ART or PrEP through a large urban health system (January 2021–September 2023) in the Northeastern United States. We used electronic health record data for PrEP and ART to examine group differences between those on LAI or oral medications using analysis of variance, chi-square tests, or Fisher exact tests. Bivariate logistic regression modeled associations between LAI ART or LAI PrEP and social determinants of health.

**Results:**

In the PrEP group, 238 patients were prescribed LAI (n = 63) or oral (n = 193) PrEP. Most PrEP patients were men (80.7%), non-Hispanic (79.5%), and White (60.7%) and had public insurance (83.1%). Compared with patients on oral PrEP, those on LAI less often experienced food insecurity, financial strain, depression, anxiety, or substance use disorders. In bivariate models, LAI PrEP inversely correlated with female sex, current smoking, depression, anxiety, and substance use disorders. In the treatment group, 1194 patients were prescribed LAI (n = 76) or oral (n = 1118) ART, with a median age of 57.0 years; 63.6% were from minoritized groups. Only age was significantly associated with LAI ART (odds ratio, 0.97; 95% CI, 0.961–0.993; *P* = .005).

**Conclusions:**

In this large retrospective cohort of patients on LAI PrEP and ART, patients receiving LAI less often experienced social barriers to accessing care. Public health interventions are needed to overcome health inequities tied to access of LAI ART for HIV prevention and treatment.

## Teaser

Long-acting injectable antiretrovirals for HIV treatment and prevention (PrEP) can improve HIV care, but data is limited on implementation in real-world clinical settings. We characterized “early adopters” of injectable ART and PrEP in terms of social determinants of health.

Long-acting injectable (LAI) antiretrovirals for HIV treatment (ART) and prevention (PrEP) are potential game-changers for the field. As opposed to daily oral ART or PrEP, which has a high bar for optimal patient adherence, LAI ART only needs to be administered 6 to 12 times per year [1–3]. For many patients, alleviation of pill fatigue and reduced stigma offset the cost of LAI medications and the potential time and energy costs of having to travel to a clinic for medication administration [2–5].

In clinical trials, LAI ART and PrEP demonstrated efficacy at treating and preventing HIV, respectively, and emerging data suggest that LAI ART may even be superior to oral ART in the patients for whom adherence is the most problematic [6]. Characteristics of early adopters of LAI ART in Italy mirrored those of large randomized clinical trial participants: Individuals were predominantly men, White, young, and had a body mass index <30. In the United States, however, people with minoritized gender and racial identities who shoulder the disproportionate burden of the HIV epidemic are under-represented in clinical trials of LAI ART for HIV treatment and prevention [7]. As LAI implementation rolls out, the overall efficacy (and population impact) will be shaped by complex social and structural determinants of health [3, 8–11]. In this early stage of implementation, it is important to understand who has access to LAI for HIV treatment and prevention so that it can be delivered in a way that promotes health equity.

This study aims to understand the early real-world clinic experience with LAI antiretrovirals, including cabotegravir/rilpivirine (CAB/RPV) for HIV treatment (ART) and cabotegravir (CAB) for HIV prevention (PrEP) using data from a large urban health system. We focus on the first year of rollout to understand the characteristics of patients who successfully initiated LAI PrEP and LAI ART as “early adopters.”

## METHODS

### Study Setting

This study took place within a large urban health system in the Northeastern United States that houses two Ryan White–funded clinics for HIV treatment and prevention. To transition patients from oral ART or PrEP to LAI, clinicians refer their patients to the on-site pharmacist as part of a collaborative drug treatment model (CDTM). CDTMs had already been in use within the health system for management of other conditions (eg, diabetes, hypertension, smoking cessation), supported by an abundance of national data that CDTMs are effective. The CDTM was expanded to include LAI PrEP and ART when it became available. Only patients previously on oral PrEP or ART are referred to pharmacists for evaluation and potential transition to LAI formulations. The pharmacist then meets with the patient, usually by phone or video visit, to verify that there are no contraindications to LAI PrEP or ART based on the Food and Drug Administration (FDA) label (prior hypersensitivity reaction to components, co-administered anticonvulsants or antimycobacterials that interact), to check for potential drug–drug interactions, to verify insurance coverage, to discuss with the patient the risks and potential benefits of LAI PrEP or ART, to assess the patient's interest in starting LAI PrEP or ART, to schedule the first injection with the clinic nurse, and to support adherence thereafter. Pharmacists follow a standardized workflow and note template in the electronic health record.

### Data Extraction Procedures

**Figure 1.**
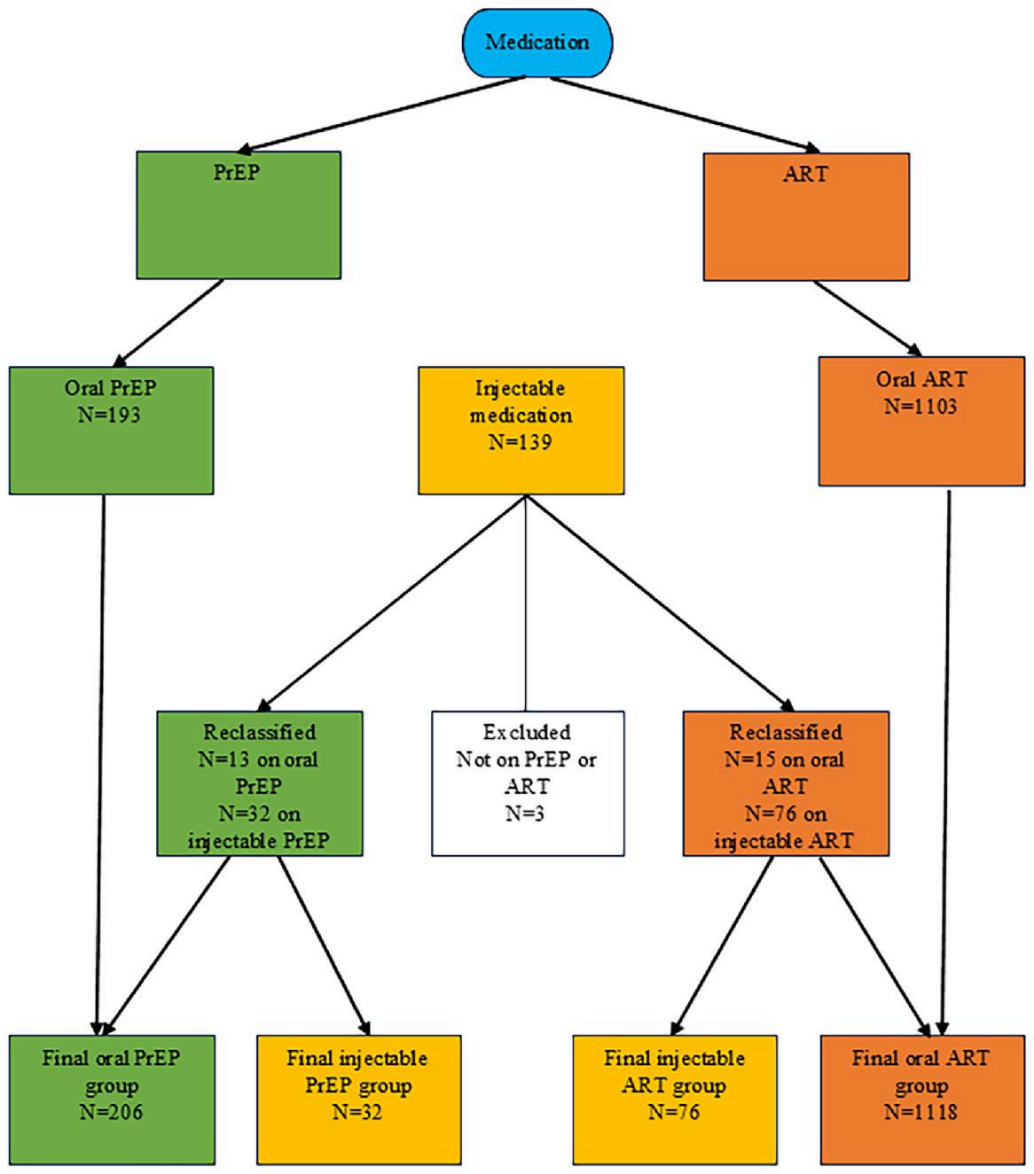
Flowchart of inclusion and exclusion of patients in the study. Abbreviations: ART, antiretroviral therapy; PrEP, preexposure prophylaxis.

In collaboration with the Joint Data Analytics Team, we conducted a retrospective cohort study using de-identified electronic health record (EHR) data of patients who were prescribed PrEP and ART from January 2021 to September 2023. We selected these dates to reflect the first full year of LAI PrEP and ART implementation. CAB/RPV for LAI ART was FDA-approved in January 2021, and the first patient in our system received it in September 2021; CAB for LAI PrEP was FDA-approved in December 2021, and the first patient in our system received it in August 2022, with the first full year of rollout ending September 2023. The study protocol was reviewed by the Institutional Review Board (IRB) at Yale University and deemed exempt (HIC #2000035174). Extracted data included demographic characteristics, patient-reported data entered by medical assistants during the clinic visit in the “Rooming” tab, medication lists with administration dates for LAI, problem lists, and HIV viral loads and dates. An independent reviewer with a clinical background reviewed the problem list for keywords; the complete list of search terms is shown in Supplementary Table 1. We generated 3 separate data sets: (1) patients on oral PrEP (green), (2) patients on oral ART (orange), and (3) patients on LAI medication (yellow) (Figure 1). Patients in the third group were separated by whether they were receiving LAI ART or PrEP, and some were reclassified as being on oral ART or PrEP if they never received an injection. Because this study was retrospective and only de-identified data were provided, patients were classified by prescription type; we did not have information on why patients were referred to the pharmacists for evaluation but ultimately did not start injectables. There were no patients in the cohort with co-administered medications (anticonvulsants or antimycobacterials) that would have been a contraindication to LAI ART or PrEP.

### Measures

The dependent variable of interest was receipt of LAI ART or LAI PrEP medication. We defined initiation as the patient receiving at least one dose of an LAI medication.

Demographic characteristics were age (continuous variable), sex (male, female), ethnicity (stratified as Hispanic or Non-Hispanic), and race (stratified as White, Black or African American, Asian, other).

Social determinants of health (SDOH) variables of interest were sourced from the “Rooming” tab of the EHR, including type of medical insurance (private, public, none), current housing (yes, yes but worried about the future, no), food insecurity (yes, no), intimate partner violence (yes, no), financial strain (hard, not hard), and available transportation to attend medical appointments (yes, no). Food insecurity is measured with a single screening item (“Within the past 12 months, you worried that your food would run out before you got the money to buy more”), with the following response options: “never true,” “sometimes true,” or “often true.” We coded “sometimes true” and “often true” as “yes” and “never true” as “no” for simplicity of analysis. For the intimate partner violence (IPV) variable, patients were listed as experiencing IPV if they answered yes to any questions in the HARK (humiliation, afraid, rape, or kicking) instrument; they were listed as not experiencing IPV if they said no to all 4 questions [12]. We also created a composite variable for SDOH issues (yes, no) that included any of the above potential barriers to care.

Comorbid conditions included any other chronic conditions (yes, no) on the problem list and self-reported smoking status (current smoker, former smoker, never smoker) in “Rooming.” Patients were categorized as having depression if they scored ≥3 on the Patient Health Questionnaire-2 (PHQ-2) reported in “Rooming” [13] or if a depressive disorder was on their problem list. Anxiety was defined by screening in “Rooming” and categorized as yes (very anxious, anxious to some extent, rather) or no (not anxious at all, only a little), or if an anxiety disorder was on their problem list. Patients were categorized as having a psychiatric disorder or substance use disorder (respectively) if any psychiatric disorder or substance use disorder was on their problem list. Unhealthy alcohol use was defined by responses to the Alcohol Use Disorders Identification Test (AUDIT) in the “Rooming” tab, and total scores were calculated using standardized methods [14]. Unhealthy alcohol use was defined by a total score of ≥4 for men and ≥3 for women [14]. Additionally, patients were categorized as having unhealthy alcohol use if an alcohol use disorder was on their problem list.

HIV viral suppression for the treatment group was defined by whether HIV was detected in the most recent viral load and recorded as a binary outcome (no if ≥20 copies/mL or yes if <20 copies/mL).

### Statistical Analysis

All data analyses were conducted using SAS, version 9.4 (SAS Institute, Inc., Cary, NC, USA). Data analyses for the PrEP group and the ART group were conducted separately. We conducted a descriptive analysis of demographic characteristics, SDOH, and comorbid conditions for each group. Means and standard deviations were calculated for normally distributed variables; medians and interquartile ranges (IQRs) was reported for non–normally distributed variables. We compared the oral and LAI groups using analysis of variance (continuous variables) and chi-square or Fisher exact tests for categorical variables. We used bivariate logistic regression to model associations between each independent variable and the outcome of prescribed LAI medication. A *P* value of <.05 defined statistical significance. Multivariable logistic regression could not be employed due to the small sample size for the primary outcome.

## RESULTS

Table 1 displays the characteristics of patients on PrEP (n = 238). Overall, the median age (IQR) was 37.0 (31.0–50.0) years. Most patients on PrEP identified as men (80.7%), non-Hispanic (79.5%), and White (60.7%). Most had public health insurance (83.1%), and almost half were experiencing housing instability or homelessness, food insecurity, or financial strain. Approximately one-third of patients on PrEP had at least 1 chronic condition. Concurrent psychiatric and substance use disorders, including unhealthy alcohol use, were prevalent.

**Table 1.** Description of Study Sample for HIV Prevention (PrEP), by Injectable vs Oral Medication^a^

Characteristic	Total PrEP Patients (n = 238)	Injectable (n = 32)	Oral (n = 206)	*P* Value
Demographic characteristics				
Median age (IQR), y	n = 238	n = 32	n = 206	.474 (DF = 1)
…	37.0 (31.0–50.0)	34.5 (30.0–49.0)	37.0 (31.0–50.0)	
Sex	n = 238	n = 32	n = 206	.013 (DF = 1)
Male	192 (80.7)	31 (96.9)	161 (78.2)	
Female	46 (19.3)	1 (3.1)	45 (21.8)	
Ethnicity	n = 230	n = 30	n = 199	.939 (DF = 1)
Hispanic	47 (20.5)	6 (20.0)	41 (20.6)	
Non-Hispanic	182 (79.5)	24 (80.0)	158 (79.4)	
Race^b^	n = 229	n = 29	n = 200	.062 (DF = 3)^c^
White	139 (60.7)	13 (44.8)	126 (63.0)	
Black/African American	54 (23.6)	11 (37.9)	43 (21.5)	
Asian	11 (4.8)	3 (10.3)	8 (4.0)	
Other	25 (10.9)	2 (6.9)	23 (11.5)	
Social determinants of Health (SDOH)				
Any SDOH issue	n = 238	n = 32	n = 206	.339 (DF = 1)
Yes	77 (32.4)	8 (25.0)	69 (66.5)	
No	161 (67.7)	24 (75.0)	137 (33.5)	
Type of insurance^b^	n = 231	n = 32	n = 199	.831 (DF = 2)^c^
Private	33 (14.3)	5 (15.6)	28 (14.1)	
Public	192 (83.1)	27 (84.4)	165 (82.9)	
No insurance	6 (2.6)	0 (0.0)	6 (3.0)	
Current housing^b^	n = 78	n = 7	n = 71	.053 (DF = 2)^c^
Yes	43 (55.1)	7 (100.0)	36 (50.7)	
Yes but worried	6 (7.7)	0 (0.0)	6 (8.5)	
No	29 (37.2)	0 (0.0)	29 (38.0)	
Food insecurity^b^	n = 57	n = 6	n = 51	.027 (DF = 1)
Yes	26 (45.6)	0 (0.0)	26 (40.6)	
No	31 (54.4)	6 (100.0)	25 (49.0)	
Intimate partner violence^b^	n = 11	n = 2	n = 9	1.000 (DF = 1)
Yes	2 (18.2)	0 (0.0)	2 (22.2)	
No	9 (81.8)	2 (100.0)	7 (77.8)	
Financial strain^b^	n = 58	n = 7	n = 51	.004 (DF = 1)
Hard	30 (51.7)	0 (0.0)	30 (58.8)	
Not hard	28 (48.3)	7 (100.0)	21 (41.2)	
Have transportation to medical appointments^b^	n = 57	n = 8	n = 49	.179 (DF = 1)
Yes	14 (24.6)	8 (100.0)	35 (71.4)	
No	43 (75.4)	0 (0.0)	14 (28.6)	
Comorbid Conditions				
Other chronic conditions	n = 238	n = 32	n = 206	. 280 (DF = 1)
Yes	70 (29.4)	12 (37.5)	58 (28.2)	
No	168 (70.6)	20 (62.5)	148 (71.8)	
Smoking status	n = 238	n = 32	n = 206	.012 (DF = 2)
Current smoker	90 (37.8)	5 (15.6)	85 (41.3)	
Former smoker	50 (21.0)	7 (21.9)	43 (20.9)	
Never smoker	98 (41.2)	20 (62.5)	78 (37.9)	
Depression	n = 100	n = 32	n = 68	<.001 (DF = 1)
Yes	63 (63.0)	8 (25.0)	55 (80.9)	
No	37 (37.0)	24 (75.0)	13 (19.1)	
Anxiety^b^	n = 50	n = 7	n = 43	.048 (DF = 1)
Yes	47 (94.0)	5 (71.4)	42 (97.7)	
No	3 (6.0)	2 (28.6)	2 (2.3)	
Psychiatric disorders	n = 238	n = 32	n = 206	.687 (DF = 1)
Yes	112 (47.1)	14 (43.8)	98 (47.6)	
No	126 (52.9)	18 (56.3)	108 (52.4)	
Substance use disorder	n = 238	n = 32	n = 206	.002 (DF = 1)
Yes	86 (36.1)	4 (12.5)	82 (39.8)	
No	152 (63.9)	28 (87.5)	124 (60.2)	
Unhealthy alcohol use^b^	n = 72	n = 5	n = 67	.182 (DF = 1)
Yes	38 (52.8)	1 (20.0)	37 (55.2)	
No	34 (47.2)	4 (80.0)	30 (44.8)	

Abbreviations: DF, degrees of freedom; IQR, interquartile range; PrEP, preexposure prophylaxis; SDOH, social determinants of health.

^a^Table values are median (IQR) for age and No. (column %) for categorical variables.

^b^Fisher exact test.

^c^One-sided probability for Fisher exact test.

There were statistically significant differences in sex, food insecurity, financial strain, smoking status, depression, anxiety, and substance use disorders between patients on LAI and oral PrEP (Table 1). Compared with patients on oral PrEP, those on LAI PrEP were more often men (96.9% vs 78.2%; *P*  *=* .013) and less likely to be food insecure (0 vs 40.6%; *P* = .027) or to have experienced financial strain (0 vs 58.8%; *P*  *=* .004). Patients on LAI PrEP also had fewer comorbid substance use and psychiatric disorders than patients on oral PrEP, including current smoking (15.6% vs 41.3%; *P* = .012), depression (25.0% vs 80.9%; *P* < .001), anxiety (71.4% vs 97.7%; *P* = .048), and substance use disorders (12.5% vs 39.8%).

In bivariate models of LAI PrEP (Table 2), the odds of women being on LAI PrEP medication were 88.5% lower (odds ratio [OR], 0.115; 95% CI, 0.015–0.869; *P* = .036) compared with the odds of men being on LAI PrEP medication. Similarly, the odds of patients who were current smokers being placed on LAI PrEP medication were 77.1% lower (OR, 0.229; 95% CI, 0.082–0.641; *P* = .005) than the odds of patients who never smoked. Compared with patients without depression, patients with depression had 92.1% lower odds (OR, 0.079; 95% CI, 0.029–0.215; *P* < .001) of being on LAI PrEP, and patients with anxiety had 94.0% lower odds (OR, 0.060; 95% CI, 0.005–0.780; *P* = .032) of being on LAI PrEP. Compared with patients without a substance use disorder, patients with a substance use disorder had 78.4% lower odds (OR, 0.216; 95% CI, 0.073–0.639; *P* = .006) of being on LAI PrEP.

**Table 2.** Bivariate Correlates of Injectable HIV Prevention (PrEP) Medication Usage Among Study Sample (n = 238)

Characteristic	Unadjusted OR (95% CI)	*P* Value
Demographic characteristics		
Age, y	0.988 (0.957–1.020)	.473
Sex	…	**…**
Male	Reference	**…**
Female	0.115 (0.015–0.869)	.036
Ethnicity	…	…
Non-Hispanic	Reference	…
Hispanic	0.964 (0.370–2.512)	.940
Race	…	.437
White	Reference	…
Black	2.479 (1.034–5.944)	.0418
Asian	3.635 (0.857–15.412)	.0799
Other	0.843 (0.178–3.985)	.8292
Social determinants of Health (SDOH)		
Any SDOH issue	…	…
No	Reference	…
Yes	0.662 (0.283–1.550)	.342
Type of insurance	…	.543
No insurance	Reference	…
Public	>999.999 (<0.001−>999.999)	.968
Private	>999.999 (<0.001−>999.999)	.968
Current housing	…	.183
No	Reference	…
Yes	1.000 (<0.001−>999.999)	.942
Yes but worried	>999.999 (<0.001−>999.999)	1.000
Food insecurity	…	…
No	Reference	…
Yes	<0.001 (<0.001−>999.999)	.938
Intimate partner violence	…	…
No	Reference	…
Yes	<0.001 (<0.001−>999.999)	.968
Financial strain	…	…
Not hard	Reference	…
Hard	<0.001 (<0.001−>999.999)	.953
Have transportation to medical appointments	…	…
No	Reference	…
Yes	<0.001 (<0.001−>999.999)	.950
Comorbid conditions		
Other chronic conditions	…	…
No	Reference	…
Yes	1.531 (0.704–3.331)	.283
Smoking status	…	.004
Never smoker	Reference	**…**
Former smoker	0.635 (0.249–1.622)	.342
Current smoker	0.229 (0.082–0.641)	.005
Depression	…	…
No	Reference	…
Yes	0.079 (0.029–0.215)	<.001
Anxiety	…	…
No	Reference	…
Yes	0.060 (0.005–0.780)	.032
Psychiatric disorders	…	…
No	Reference	…
Yes	0.857 (0.405–1.815)	.687
Substance use disorder	…	…
No	Reference	…
Yes	0.216 (0.073–0.639)	.006
Unhealthy alcohol use	…	…
No	Reference	…
Yes	0.203 (0.022–1.911)	.163

Abbreviations: OR, odds ratio; PrEP, preexposure prophylaxis; SDOH, social determinants of health.

Table 3 shows the characteristics of patients on ART (n = 1194), who had a median age (IQR) of 57.0 (43.0–64.0) years. Most patients on ART identified as male (65.8%) and non-Hispanic (79.1%); almost half of patients on ART were Black (49.3%). Most patients on ART had public insurance (82.2%) and stable housing (88.7%); approximately one-quarter had food insecurity or financial strain. As would be expected of a large HIV primary care clinic population, approximately half of patients on ART had at least 1 chronic condition or psychiatric disorder. Substance use disorders were diagnosed in 33.4% of patients, and 52.7% had diagnosed unhealthy alcohol use. For most (78.8%) patients on ART, the most recent HIV viral load was undetectable.

**Table 3.** **Description of Study Sample for HIV Treatment (ART), by Injectable vs Oral Medication** ^a^

Characteristic	Total ART Patients (n = 1194)	Injectable (n = 76)	Oral (n = 1118)	*P* Value
Demographic characteristics				
Median age (IQR), y	n = 1194	n = 76	n = 1118	.004 (DF = 1)
…	57.0 (43.0–64.0)	50.0 (37.0–60.0)	58.0 (44.0–64.0)	
Sex	n = 1194	n = 76	n = 1118	.623 (DF = 1)
Male	785 (65.8)	48 (63.2)	737 (65.9)	
Female	409 (34.3)	28 (36.8)	381 (34.1)	
Ethnicity	n = 1179	n = 74	n = 1105	.882 (DF = 1)
Hispanic	247 (21.0)	15 (20.3)	232 (21.0)	
Non-Hispanic	932 (79.1)	59 (79.7)	873 (79.0)	
Race	n = 1135	n = 74	n = 1061	.320 (DF = 3)
White	407 (36.4)	32 (43.2)	375 (35.3)	
Black/African American	559 (49.3)	35 (47.3)	524 (49.4)	
Asian	21 (1.9)	0 (0.0)	21 (2.0)	
Other	148 (13.0)	7 (9.5)	141 (13.3)	
Social determinants of Health (SDOH)				
Any SDOH issue	n = 1194	n = 76	n = 1118	.189 (DF = 1)
Yes	478 (40.3)	25 (32.9)	453 (40.5)	
No	716 (60.0)	51 (67.1)	665 (59.5)	
Type of insurance	n = 1152	n = 75	n = 1077	.108 (DF = 2)
Private	160 (13.9)	14 (18.7)	146 (13.6)	
Public	947 (82.2)	61 (81.3)	886 (82.3)	
No insurance	45 (3.9)	0 (0.0)	45 (4.2)	
Current housing^b^	n = 467	n = 22	n = 445	.103 (DF = 2)^c^
Yes	414 (88.7)	18 (81.8)	396 (88.9)	
Yes but worried	11 (2.4)	2 (9.1)	9 (2.0)	
No	42 (9.0)	2 (9.1)	40 (9.0)	
Food insecurity^b^	n = 402	n = 23	n = 379	.870 (DF = 1)
Yes	91 (22.8)	5 (21.7)	88 (23.2)	
No	309 (77.3)	18 (78.3)	291 (76.8)	
Intimate partner violence^b^	n = 75	n = 3	n = 72	.602 (DF = 1)
Yes	6 (8.0)	0 (0.0)	6 (8.3)	
No	69 (92.0)	3 (100.0)	66 (91.7)	
Financial strain	n = 415	n = 22	n = 393	.366 (DF = 1)
Hard	116 (28.0)	8 (36.3)	108 (27.5)	
Not hard	299 (72.1)	14 (63.6)	285 (72.5)	
Have transportation to medical appointments^b^	n = 432	n = 25	n = 407	1.000 (DF = 1)
Yes	37 (8.6)	2 (8.0)	35 (8.6)	
No	395 (91.4)	23 (92.0)	372 (91.4)	
Comorbid conditions				
Other chronic conditions	n = 1194	n = 76	n = 1118	.454 (DF = 1)
Yes	662 (55.4)	39 (51.3)	623 (55.7)	
No	532 (44.6)	37 (48.7)	495 (44.3)	
Smoking status	n = 1193	n = 76	n = 1117	.804 (DF = 2)
Current smoker	337 (28.3)	19 (25.0)	318 (28.5)	
Former smoker	429 (36.0)	29 (38.2)	400 (35.80	
Never smoker	427 (35.8)	28 (36.8)	399 (35.7)	
Depression	n = 1139	n = 74	n = 1065	.880 (DF = 1)
Yes	394 (34.6)	25 (33.8)	369 (34.7)	
No	745 (65.4)	49 (66.2)	696 (625.4)	
Anxiety^b^	n = 264	n = 16	n = 242	.593 (DF = 1)
Yes	250 (94.7)	15 (93.8)	235 (94.8)	
No	14 (5.3)	1 (6.3)	13 (5.2)	
Psychiatric disorders	n = 1194	n = 76	n = 1118	.296 (DF = 1)
Yes	572 (47.9)	32 (42.1)	540 (48.3)	
No	622 (52.1)	44 (57.9)	578 (51.7)	
Substance use disorder	n = 1194	n = 76	n = 1118	.547 (DF = 1)
Yes	399 (33.4)	23 (30.3)	376 (33.6)	
No	795 (66.6)	53(69.7)	742 (66.4)	
Unhealthy alcohol use	n = 349	n = 20	n = 329	.502 (DF = 1)
Yes	184 (52.7)	12 (40.0)	157 (47.7)	
No	165 (47.3)	8 (60.0)	172 (52.3)	
HIV viral suppression	n = 1167	n = 76	n = 1091	.135 (DF = 1)
No (≥20 copies/mL)	248 (21.3)	11 (14.5)	237 (21.7)	
Yes (<20 copies/mL)	919 (78.8)	65 (85.5)	854 (78.3)	

Abbreviations: ART, antiretroviral therapy; DF, degrees of freedom; IQR, interquartile range; PrEP, preexposure prophylaxis; SDOH, social determinants of health.

^a^Table values are median (IQR) for age and No. (column %) for categorical variables.

^b^Fisher exact test.

^c^One-sided probability for Fisher exact test.

The only statistically significant difference between patients on LAI and oral ART was age (LAI: median [IQR], 50.0 [37.0–60.0] years; vs oral: median [IQR], 58.0 [44.0–64.0] years) Similarly, in bivariate models of LAI ART, only age had a statistically significant association, such that every 1-year increase in age was associated with a 2.30% decrease (OR, 0.97; 95% CI, 0.961–0.993; *P* = .005) in odds of receiving LAI ART.

## DISCUSSION

To our knowledge, this is the first study to compare the sociodemographic characteristics of patients on LAI PrEP and LAI ART with those of patients on oral PrEP and oral ART, respectively, in a large health system in the United States. We found significant differences in sex, food insecurity, financial strain, smoking status, depression, anxiety, and substance use disorders between patients on oral PrEP and LAI PrEP. However, statistically significant differences were only found in age between patients on LAI ART and oral ART. Overall, our findings suggest that patients with fewer medical, psychiatric, and social comorbidities are more likely to be placed on LAI medication for HIV prevention, though this trend was not observed for LAI use in HIV treatment.

In this sample, compared with patients on oral PrEP, those on LAI PrEP were less likely to experience food insecurity and financial strain. Patients on LAI PrEP were also less likely to have diagnosed depression or anxiety, to be currently smoking, or to have diagnosed substance use disorders. Although PrEP is covered under the Connecticut AIDS Drug Assistance Program (CADAP) for those who cannot afford it [15], health disparities seem to persist, at least for “early adopters” of LAI PrEP. Reasons for this inequity could be due to patients' lack of awareness of LAI medications, healthcare providers not engaging in shared clinical decision-making, or providers not recommending patients they see as more “unstable” for LAI medications. It is possible that patients are selectively referred to the pharmacists for LAI initiation in the same way that clinicians may selectively prescribe LAI PrEP for the patients they perceive to be most likely to adhere. Even if patients are aware of LAI PrEP, they may have legitimate concerns about receiving injections, the cost of traveling to the clinic to receive injections (financial and time), or relinquishing autonomy of medication administration [16, 17]. There may be important differences in cost considerations for patients who are privately insured or on Medicare (where LAI is covered through medical benefits) and those who are on Medicaid (where LAI is covered through pharmacy benefits), though recently updated federal guidance requires coverage of all three forms of PrEP by insurers without cost sharing [18]. In prior studies, patients cited concerns about the safety and long-term side effects of LAI PrEP as reasons for not switching [16, 17, 19], while providers have cited low uptake among patients during shared clinical decision-making, issues with workflow for lab work, and difficulties procuring LAI medication as barriers to implementation [19]. Other prior research highlights patient preference for LAI PrEP due to the convenience and privacy it offers compared with oral PrEP, when having a pill bottle may inadvertently “out” the patient [20]. Education and system-level supports may also be critical factors that determine successful initiation of LAI treatment, as patients must navigate complex insurance forms, deductibles, and other logistical challenges, which may be influenced by their understanding of and access to support resources.

In this early evaluation of LAI implementation, only 6.3% of ART patients in our sample were on LAI ART. On average, the median age of patients on LAI ART was 8 years younger than the median age of patients on oral ART. It is not clear from our analysis whether older age was a proxy for other issues (ie, medical comorbidity, patient preference) that drove reduced LAI ART in older patients. The relatively low number of patients on LAI ART overall has been seen elsewhere, with patients citing distrust of injectables and concerns about possible side effects or long-term impacts as well as loss of autonomy with controlling their medication intake [16, 21]. Patients who switched to LAI ART reported ease of receiving a few injections every year over the burden of taking a pill every day, as well as reduced feelings of internalized stigma and daily reminders of their HIV [22–25]. Patients also reported that injectables reduced the risk of “non-desired disclosure” of their HIV status to partners or peers and lessened worries about pills or pill bottles being seen by others [26].

The current FDA label of LAI cabotegravir/rilpivirine requires that patients be virally suppressed on oral ART before switching [4, 27], which may be a barrier to access for some [8]. Adherence to oral medication may be more difficult for patients if they are experiencing substance use, mental health issues, or housing instability [4, 28–37]. A recent small study counters this narrative, demonstrating greater efficacy of LAI ART over oral ART for the patients for whom adherence is most challenging [38–40].

To be sure, LAI PrEP or ART may not be preferred over oral options by patients or clinicians for a wide variety of reasons. Yet our findings that more medically and socially stable patients are more often on LAI PrEP suggest that there may be additional hurdles to some patients accessing LAIs. Patients and clinicians should engage in shared decision-making about LAIs for either PrEP or ART to help mitigate health disparities.

## Limitations

There are several limitations to this study. First, the low number of observed cases for patients on LAIs (6.38% of ART patients and 26.5% of PrEP patients) may have reduced the statistical power of the study to detect the true association between independent variables and being placed on LAI medication. The overall low number of LAI prescriptions during the first full year of implementation may have reduced our ability to detect meaningful differences between groups. However, we intentionally selected this first year to understand characteristics of the earliest adopters before dedicated outreach programs were put in place. In this retrospective study, available EHR data depended on documentation as part of routine care. This resulted in missing data for many variables, which impacted our ability to assess the relationship between all variables of interest and the independent variable. The study took place in New England, which is generally well-resourced with Medicaid expansion, including having a dedicated pharmacist as part of a collaborative drug treatment model for LAI, which may reduce generalizability to other settings. These resources, including pharmacist services, serve to facilitate potential initiations of LAI PrEP and ART, so that the observed estimates may be even higher than in other settings where resources are less abundant. Lastly, the aggregation of comorbid chronic health conditions into a binary variable may have oversimplified clinical considerations for starting LAI.

Despite these limitations, our study also had many strengths, including being the first study, to our knowledge, to compare the sociodemographic profile of patients on LAI and oral HIV medication. Future research should facilitate shared decision-making about patient transition to LAI PrEP and LAI ART medication and reduce barriers to equitable access.

## CONCLUSIONS

We found that patients receiving LAI medication for PrEP were less likely than those on oral PrEP to experience medical, psychiatric, and social comorbidities, and patients on LAI ART were significantly younger than those on oral ART. These findings suggest that social determinants of health affect which patients can successfully access LAI PrEP and ART.

## Supplementary Material

ofaf029_Supplementary_Data

## Supplementary Data

Supplementary materials are available at *Open Forum Infectious Diseases*online. Consisting of data provided by the authors to benefit the reader, the posted materials are not copyedited and are the sole responsibility of the authors, so questions or comments should be addressed to the corresponding author.
