# Supplementary material for: Real-world Rollout of Injectable Antiretrovirals for HIV Prevention and Treatment: Correlates of Early Adoption
Source: Open Forum Infect Dis. 2025 Jan 20;12(2):ofaf029. doi: 10.1093/ofid/ofaf029 (PMC11979452; doi:10.1093/ofid/ofaf029)
Supplement: ofaf029_Supplementary_Data [file ofaf029_supplementary_data.docx]

**Supplementary Table: Search Terms Used for the Categorization of Patient Problem List**

| **Problem List Variable Name** | **Keyword Search Terms** |
| --- | --- |
| Psychiatric Disorder | Depression, Anxiety, Suicid*, Schizo*, Mood, Psychotic |
| Substance Use Disorder | Alcohol, Cocaine, Stimulant, Opioid, Substance |
| Other Chronic Conditions | Asthma, COPD, Diabetes, Hypertension, Chronic Kidney |
| SDOH | incarceration, Violence, Housing, Homeless, Food, Transport |
